# Adulteration of Food

**Published:** 1888-09

**Authors:** 


### ADULTERATION OF FOOD.

The Massachusetts Board of Health, through its Secretary, Dr. W. S. Abbott presents the following summary of the number of samples of food and drugs examined for the year ending September 30, 1887, together with the number of each sort found to be adulterated and the number free from adulteration :

| | |
|---|---|
| Number of samples of food examined | 4,870 |
| " " found to be pure | 3,163 |
| " " adulterated or not conforming to the statutes | 1,707 |
| Percentage of adulteration | 35.05 |
| Number of samples of milk (included above) | 3,081 |
| " " " above standard | 1,900 |
| " " " below standard, or otherwise adulterated | 1,181 |
| Percentage of adulteration | 38.33 |
| Number of samples of drugs | 550 |
| " " " of good quality | 400 |
| " " " not conforming to the statutes | 150 |
| Percentage of adulteration | 27.27 |
| Total examinations of food and drugs | 5,420 |
| " " of good quality | 3,563 |
| " " not conforming to the statutes | 1,857 |
| Percentage of adulterations | 34.26 |

The percentage of adulterated articles stated as having been found in the samples collected in 1887 was 29.7, being the lowest of any year since the Food and Drug Acts went into operation. The actual difference between this percentage and that of previous years is greater than the apparent difference, for the reason that in the earlier years samples of food were collected from a greater variety of articles. The experience of the past few years has enabled the board to omit many articles in the work of collection, such experience having shown that many staple articles are practically free from adulteration. The percentage of adulteration found by the analysts is, therefore, a percentage of articles liable to adulteration, and not that of the general food supply.

The different articles of food which have been collected by the inspectors from all parts of the state, and submitted to examination or analysis for the purpose of determing their quality, have been mainly those which are subject to adulteration, the previous work of this board in this department of investigation, having conclusively shown that many staple articles of food are not subject to fraud, at least, so far as their sale in this state is concerned.

The following list will show the number of samples of each different article examined.

| | | | |
|---|---|---|---|
| Allspice | 36 | Horseradish | 2 |
| Essence of almond | 4 | Jam | 3 |
| Arrowroot | 1 | Lard | 18 |
| Baking Powder | 21 | Maple sugar | 13 |
| Canned vegetables | 31 | Maple syrup | 7 |
| Bread | 1 | Mapeline | 2 |
| Butter | 104 | Macaroni | 2 |
| Cake | 1 | Molasses | 97 |
| Cayenne | 27 | Mace | 20 |
| Cassia | 103 | Mustard | 118 |
| Cinnamon | 13 | Pepper, black | 144 |
| Celery salt | 6 | Pepper, white | 70 |
| Cheese | 21 | Pimento | 12 |
| Chocolate | 5 | Pickles | 1 |
| Cider Jelly | 2 | Olive Oil | 17 |
| Cloves | 60 | Nutmeg | 5 |
| Coffee | 11 | Sage | 5 |
| Cocoa | 6 | Savory | 2 |
| Corn starch | 3 | Soda | 16 |
| Candy | 45 | Sugar of milk | 1 |
| Cream of tartar | 198 | Sugar | 8 |
| Curry powder | 2 | Powdered sugar | 6 |
| Custard powder | 1 | Thyme | 3 |
| Ginger | 123 | Tea | 28 |
| Gelatine | 6 | Vinegar | 224 |
| Ginger ale | 3 | Yeast | 8 |
| Honey | 32 | | |

From this list it will be seen that the articles chiefly selected for examination from the food markets of our state are the different sorts of spices, baking powders, cream of tartar, honey, lard, molasses and syrups, olive oil, vinegar and canned or preserved vegetables. Those which comprise the larger numbers in this list are the articles most liable to adulteration.

The actual percentage of the articles found to be adulterated in the different years of the operation of these acts (excepting 1883) is as follows :

| | | | |
|---|---|---|---|
| Percentage of adulteration, | 1884 | 54.2 | per cent. |
| " " | 1885 | 42.4 | " |
| " " | 1886 | 38.7 | " |
| " " | 1887 | 34.3 | " |

It should not be understood that these figures in any year represent the actual amount of adulteration which exists among the different articles which are under the supervision of the laws, for the reasons already stated.

The actual benefits derived from the operation of these statutes have already been alluded to in previous reports, and the estimates therein given do not require a repetition here, except to say that the actual gain to the consumer has many times exceeded the cost of execution of the laws.

The benefit is mainly an economical one, inasmuch as the forms of adulteration are chiefly of a commercial character. Injurious forms of adulteration, however, are special objects of search, and there can be no question that sanitary advantages are secured by the general operation of the law.

In certain articles of food which have been specially liable to fraud, such as the various sorts of spices, condiments, cream of tartar, etc., adulteration has been greatly diminished, and is, in many instances, limited to parties whose business is conducted outside of the state, and cannot, therefore, be easily reached except through the customary notice sent to the retailer who buys of such outside parties.

These notices have proved to be a very efficient mode of diminishing adulteration. It is not the retailer who is seriously affected by the receipt of such a notice, but the wholesale dealer or manufacturer, to whom it is speedily forwarded by the retailer, and who finds that he cannot afford to continue in wholesale imposition.

The total number of samples of food and drugs of all kinds examined during the year was 5,420, an increase of 642 over that of any previous year, the principal increase having been in the number of samples of milk. This increase was effected in order to secure a more explicit compliance with the provision of the statute requiring an expenditure of three-fifths of the appropriation in the enforcement of the laws relative to milk and milk products.

A reasonable degree of improvement in the results attained by the execution of the statutes has been accomplished, and if constant improvement from year to year in all directions is not secured, it is certainly a great gain to be able to prevent further adulteration, and to require a reasonable conformity to standards of purity and excellence. So long as avarice, carelessness, neglect and ignorance are common characteristics of mankind, some sort of inspection must be maintained as a safeguard against harm on the one hand and fraud on the other.—*The Anti-Adulteration Journal.*

